# High intensity resistance training causes muscle damage and increases biomarkers of acute kidney injury in healthy individuals

**DOI:** 10.1371/journal.pone.0205791

**Published:** 2018-11-06

**Authors:** Tania C. Spada, José M. R. D. Silva, Lucila S. Francisco, Lia J. Marçal, Leila Antonangelo, Dirce M. T. Zanetta, Luis Yu, Emmanuel A. Burdmann

**Affiliations:** 1 Division of Nephrology and LIM 12, University of São Paulo Medical School and Hospital das Clínicas, São Paulo, Brazil; 2 School of Science, São Paulo State University, Bauru, Brazil; 3 Division of Sports, University of Guarulhos, Guarulhos, Brazil; 4 Department of Pathology, Clinical Laboratory and LIM 3, Hospital das Clínicas, University of São Paulo Medical School, São Paulo, Brazil; 5 School of Public Health of the University of São Paulo, São Paulo, Brazil; Istituto Di Ricerche Farmacologiche Mario Negri, ITALY

## Abstract

**Purpose:**

High-intensity interval resistance training (HIIRT) is an increasingly popular exercise program that provides positive results with short sessions. This study aimed to evaluate whether an HIIRT session causes muscle and kidney damage.

**Methods:**

Fifty-eight healthy volunteers (median age 24 years, 50% women) participated in this study and performed a HIIRT session. The Borg CR10 scale for pain (CR10P) and blood and urine samples were collected before (baseline) and 2 and 24 hours after the HIIRT session. Blood samples were analyzed for serum creatinine (SCr), creatine kinase (CK) and myoglobin. Urine samples were assessed for creatinine, neutrophil gelatinase-associated lipocalin, interleukin 18, calbindin, microalbuminuria, trefoil factor-3 and β-2 microglobulin.

**Results:**

CR10P had a significant increase at 2 and 24 hours post-workout, and CK increased significantly at 2 hours and increased further at 24 hours. Myoglobin increased significantly at 2 hours and remained elevated at 24 hours. SCr increased modestly but significantly at 24 hours only in men. Three men met the KDIGO diagnostic criteria for acute kidney injury. The urinary kidney injury biomarkers increased significantly at 2 hours and returned to the baseline values 24 hours after HIIRT.

**Conclusions:**

A single HIIRT session caused early and significant elevations in CK, myoglobin, SCr, microalbuminuria and urinary biomarkers indicative of kidney tubular injury, suggesting the occurrence of muscle and kidney damage.

## Introduction

The adequate and regular performance of moderate-intensity exercise provides well-known health benefits. It decreases the risk of insulin resistance, metabolic syndrome and type 2-diabetes, reduces all-cause respiratory and cardiovascular mortality, reduces cardiovascular morbidity and prevents cardiac disease, decreases premature mortality risk and effectively prevents several chronic diseases [1–3].

Nevertheless, the frequency of individuals performing regular exercise remains low in developed and developing countries [4,5]. One of the main causes reported worldwide by individuals for physical inactivity is a lack of time to perform an exercise program [6].

High-intensity interval training (HIIT) has emerged as one of the fastest growing exercise programs in recent years. One of the main reasons for its popularity is because it provides benefits similar to conventional workouts with shorter training sessions. Additionally, HIIT practitioners consider it to be more dynamic and less tedious than conventional continuous exercise sessions [7,8]. Although there is not a universal definition, HIIT typically consists of short bouts of sequences of submaximum-maximum intensity exercises separated by periods of rest or low-intensity exercises [9]. Weekly sessions of HIIT improve muscle oxidative capacity and aerobic and anaerobic capacity, decrease arterial blood pressure, decrease body weight in obese patients, and improve glucose regulation and insulin resistance in type 2 diabetes [7,8]. Subsequently, high-intensity interval resistance training (HIIRT) was developed to combine the benefits of HIIT with work on muscle resistance. Accordingly, HIIRT follows the same methodology as HIIT but uses resistance exercises [10].

Nonetheless, although moderate-intensity exercises are safe, strenuous and high-intensity exercises may cause unfavorable effects, including rhabdomyolysis and acute kidney injury (AKI), and they may be deleterious to long-term health outcomes, especially in poorly trained or sedentary individuals [3]. In fact, case reports of severe rhabdomyolysis, with and without AKI, associated with different modalities of high-intensity exercises have recently been published [11–13].

Considering the growing dissemination of high-intensity interval training methods and the potential risks associated with the paucity of data on its systemic effects, this exploratory study aimed to assess if a HIIRT session causes muscle and kidney damage in young, healthy and physically active individuals.

## Methods

### Ethics

This study was approved by the Ethical Committee of the University of Sao Paulo Medical School (Comitê de Ética em Pesquisa da Faculdade de Medicina da Universidade de São Paulo, n° 156/16). All individuals were informed about the possible risks and benefits of the study, and agreed to participate and signed the informed consent form.

### Study design and participants

Undergraduate students at a Physical Education School (Universidade de Guarulhos, Guarulhos, Brazil) were invited to participate in this cohort prospective study. Inclusion criteria were age >18 years old, not having a chronic disease, regular practice of physical exercise, no use of medications or diet supplements. Exclusion criteria were inability to correctly perform the prescribed exercise and baseline serum or urinary laboratory parameters above the normal range.

### Data collection

The data collection was performed in three different visits, which are described below.

#### First visit

At the first visit, we explained to the participants the scales used for assessing effort and pain, the Borg Rating of Perceived Exertion Scale (RPE) and the Borg CR10 scale (CR10P) for pain, respectively. The RPE is a numerical scale ranging from 6 to 20 used to measure effort perception related to exercise intensity [14]. The CR10P is a numerical scale ranging from zero to 10, which was used to assess thigh pain [14].

Participants were instructed in the Tabata et al. [15] style squat exercise and performed one set of exercises to test their capacity. They received instructions about the preparation for the collection of blood and urine samples, were directed on how to eat (the individuals were instructed to drink water when thirsty and keep usual food intake, avoiding heavy or too fat meals) and were asked to not perform any exercise during the 48 hours preceding visits 2 and 3. Additionally, they were instructed to continue not taking any medication or diet supplement before and during the next visits.

We obtained height and weight for all individuals and calculated body mass index (BMI) using the formula weight/height^2^.

#### Second visit

Initially, the participants completed the CR10P scale, and then the blood and urine samples were collected. Next, the participants performed five minutes of warm-up on a cycle ergometer (Life cycle 9500HR, Life Fitness, Lake Forest, IL, USA).

After these procedures, the individuals performed four minutes of HIIRT (squat exercises in the Tabata style). The HIIRT session consisted of eight sets of squats performed with the fastest speed and highest number of repetitions achievable for 20 seconds with 10 seconds of rest between sets. Immediately after the exercise, the participants completed the RPE and CR10P scales.

After the exercise, the participants drank 300 ml of tap water and rested for two hours. This amount of water was given after the exercise because it was considered sufficient to replace losses during the 4 minutes of exercise and to allow an adequate urinary output in order to collect the sample. Then, a CR10P scale was completed, and blood and urine samples were collected again.

#### Third visit

Twenty-four hours after the exercise, a CR10P scale was completed, and blood and urine samples were collected.

#### Sample collection

Blood was collected in 5 ml vials with separator gel and no EDTA. Blood samples were centrifuged at 4,500 rpm for 20 minutes at 4°C. Serum was used for the assessment of serum creatinine (SCr), creatine phosphokinase (CK) and myoglobin. Fifteen milliliters of each urine sample was transferred to Falcon vials that were centrifuged at 4,500 rpm for 20 minutes at 4°C; the urine was then divided into two aliquots and saved immediately at -70°C for later assessment of urinary biomarkers of renal injury. The remainder of the urine was used to measure creatinine.

#### Laboratory dosages

Creatinine was assessed using the Jaffé method (Cobas 8000 modular, ROCHE Diagnostics, Indianapolis, IN, USA), CK using the UV test and myoglobin using the electrochemiluminescence method.

Urinary neutrophil gelatinase-associated lipocalin (UNGAL), urinary interleukin 18 (IL-18), calbindin, microalbuminuria (μalbum), trefoil factor-3 (TFF3) and β-2 microglobulin (β2M) were assessed with a Luminex xMAP (Bio-plex PRO II Wash Station, MAG-PIX, Millipore Corporation, Darmstadt, Germany).

### Statistical analysis

Results were evaluated using the Kolmogorov-Smirnov test. The mean and standard deviation were used for variables with a normal distribution. The median (with the 25% and 75% quartiles) was used for variables without a normal distribution. Data were analyzed by the bi-caudal unpaired Student t-test, the nonparametric repeated measures ANOVA followed by Dunn’s multiple post-test or by repeated measures analysis of variance followed by the Tukey-Kramer multiple comparisons post-test, as appropriate. Correlations between RPE and muscle and renal biomarkers were assessed by bi-caudal Spearman Rank Correlation. Statistical significance was set at p<0.05.

## Results

### Study participants

**Fig 1:**
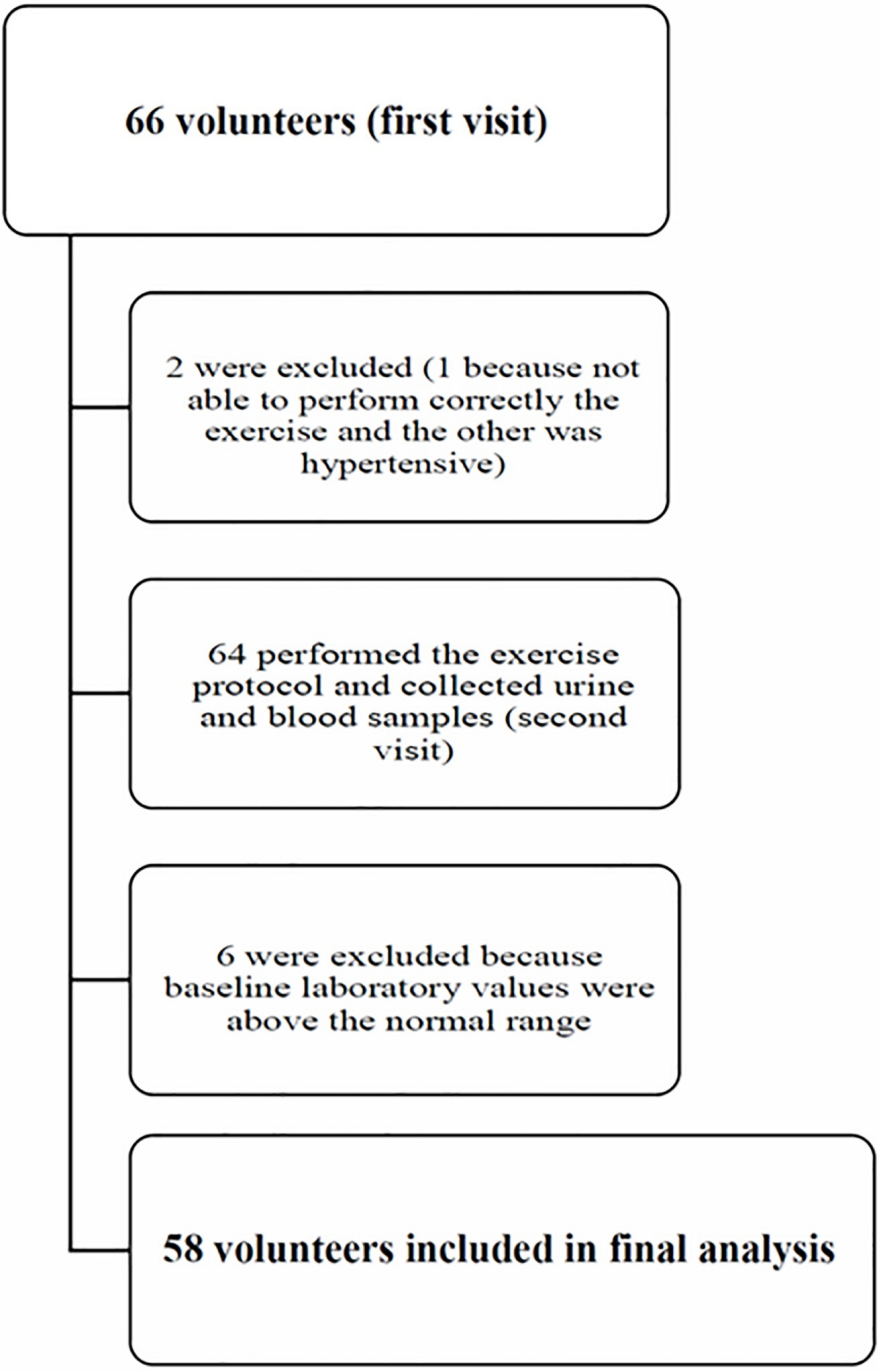
Enrollment chart of volunteers in the study.

Sixty-six individuals agreed to participate. They had been performing regularly different modalities of aerobic and anaerobic exercises during their school classes and personal trainings, mostly strength training. Eight individuals were excluded, one because was not able to correctly perform the exercise, other denied chronic diseases at study admission but was hypertensive before performing the exercise and was therefore excluded, and six because they had baseline laboratory values (before performing the exercise) above the normal range. The final sample for analysis comprised 58 individuals (Fig 1), 29 men and 29 women, with an age of 24 years (21–28 years) and a BMI of 24.5±2.8 (Table 1). BMI was similar for the men and women.

**Table 1:** Characteristics of the study participants.

	Men (n = 29)	Women (n = 29)	All (n = 58)
Age	24 (22–28)	24 (21–28)	24 (21–28)
Weight (kg)	77.3±12.0	61.3±9.7	69.3±13.5
Height (cm)	176 (172–182)	163 (158 a 165)	168 (162–176)
BMI (kg/m^2)^	24.5±2.8	23.3±3.4	23.9±3.1

Data are the median (25% and 75% quartiles) or mean±SD; BMI: body mass index.

### Borg scales

The RPE was 15 (15–18), indicating a hard effort. The results of the CR10P scale for pain increased significantly from zero at pre-exercise (0–1) to 1.5 at two hours post-exercise (0–3), p<0.001 vs. pre-exercise and to 4 at 24 hours post-exercise (2–5), p<0.001 vs. pre-exercise and vs. two hours. The RPE and CR10P scale results were both similar in men and women.

### Muscular injury markers

**Fig 2:**
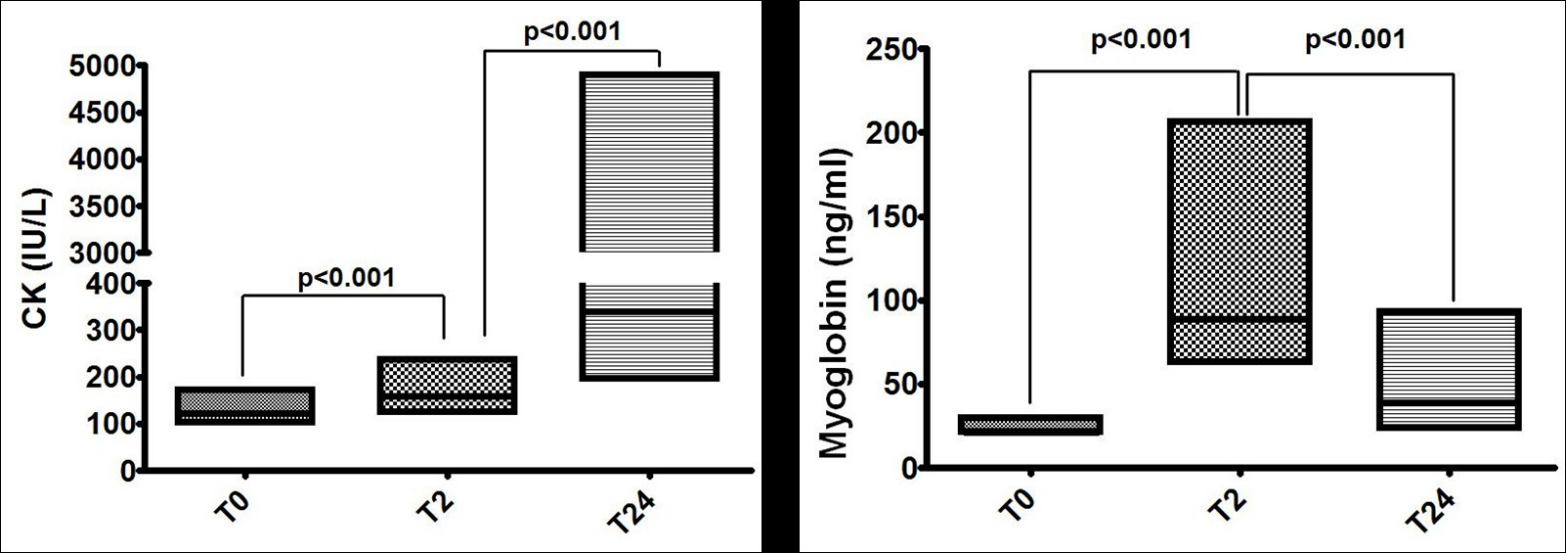
Box plots (median, 25% and 75% quartiles) of biomarkers of muscle damage: Creatine phosphokinase (CK) and myoglobin.

A statistically significant CK increase occurred after exercise, with CK values almost three times over the baseline after 24 hours (Fig 2). Among the 58 individuals, 69% increased CK values over the reference range limits. In addition, in 19% of the individuals CK values (IU/L) increased more than five times above the normal range, from 167 (139–306, 25–75% quartiles) to 1,537 (1,177–4,559, 25–75% quartiles), p<0.0001.

Serum myoglobin increased significantly two hours after the exercise and remained significantly elevated 24 hours after exercise (Fig 2 and Table 2). Among the 58 individuals 74% increased serum myoglobin above the reference range limits after exercise. These changes were similar in both men and women.

**Table 2:** Assessment of muscle and kidney injury biomarkers at baseline and at 2 and 24 hours after exercise.

	Baseline (T0)	2 hours after HIIRT (T2)	24 hours after HIIRT (T24)	p
**Muscle injury markers**				
CK (IU/L)	123 [102–172]	158 [124–238]^a^	340 [196–4906]^b^^,^^c^	<0.0001
Myoglobin (ng/ml)	21.0 [21.0–29.8]	88.6 [62.9–206.6]^a^	39.1 [23.6–93.6]^b^	<0.0001
**Serum kidney injury marker**				
SCr (mg/dl)	0.91±0.17	0.90±0.17	0.94±0.23^d^	0.0274
**Urine kidney injury markers**				
UNGAL (ng/mgCr)	18.5 [8.9–33.7])	33.4 [16.6–49.7]^e^	15.3 [6.8–47.2]	<0.0001
IL-18 (ng/mgCr)	0.014 [0.007–0.029]	0.026 [0.015–0.062^a^	0.015 [0.016–0.043]	0.0003
μalbumin (μg/mgCr)	4.3 [3.0–9.8]	20.0 [7.2–29.9] ^a^	3.2 [1.6–6.3]	<0.0001
Calbindin (ng/mgCr)	27.4 [11.1–52.6]	54.8 [30.9–88.1] ^a^	36.8 [21.2–59.9]	0.0003
TFF3 (ng/mgCr)	347 [234–5162]	508 [357–719] ^a^	339 ([210–531]	<0.0001
β2M (ng/mgCr)	80 [52–107]	164 [76–327] ^a^	63 [36–82]	<0.0001

Values are the mean±SD or median (25–75% quartiles); HIIRT: high-intensity resistance training; RPE: rating of perceived exertion; CK: creatine phosphokinase; SCr: serum creatinine; UNGAL: urinary neutrophil gelatinase-associated lipocalin; IL-18: interleukin 18; TFF3: trefoil factor-3; β2M: β-2 microglobulin.

^a^ vs. T0 p<0.001

^b^ vs. T0 p<0.001

^c^ vs. T2 p<0.001

^d^ vs. T2 p<0.05

^e^ vs. T0 p<0.01.

### Serum renal biomarkers

SCr increased slightly but significantly 24 hours after the exercise in 47% of the individuals and in 6% the elevation was compatible with AKI definition (Table 2).

When men and women were analyzed separately, we found that SCr increased significantly in men (baseline 1.03±0.13 mg/dl, two hours 1.03±0.13 mg/dl and 24 hours 1.10±0.20 mg/dl, p<0.05) but was steady in women (baseline 0.79±0.12 mg/dl, two hours 0.77±0.10 mg/dl and 24 hours 0.77±0.10 mg/dl, p>0.05). The SCr of two men increased by more than 0.3 mg/dl from the baseline level at 24 hours after the exercise (from 1.19 mg/dl to 1.7 mg/dl and 1.21 mg/dl to 1.63 mg/dl), and the SCr of one man increased by 0.27 mg from baseline to 24 hours (0.96 mg/dl to 1.23 mg/dl).

### Urinary renal biomarkers

**Fig 3:**
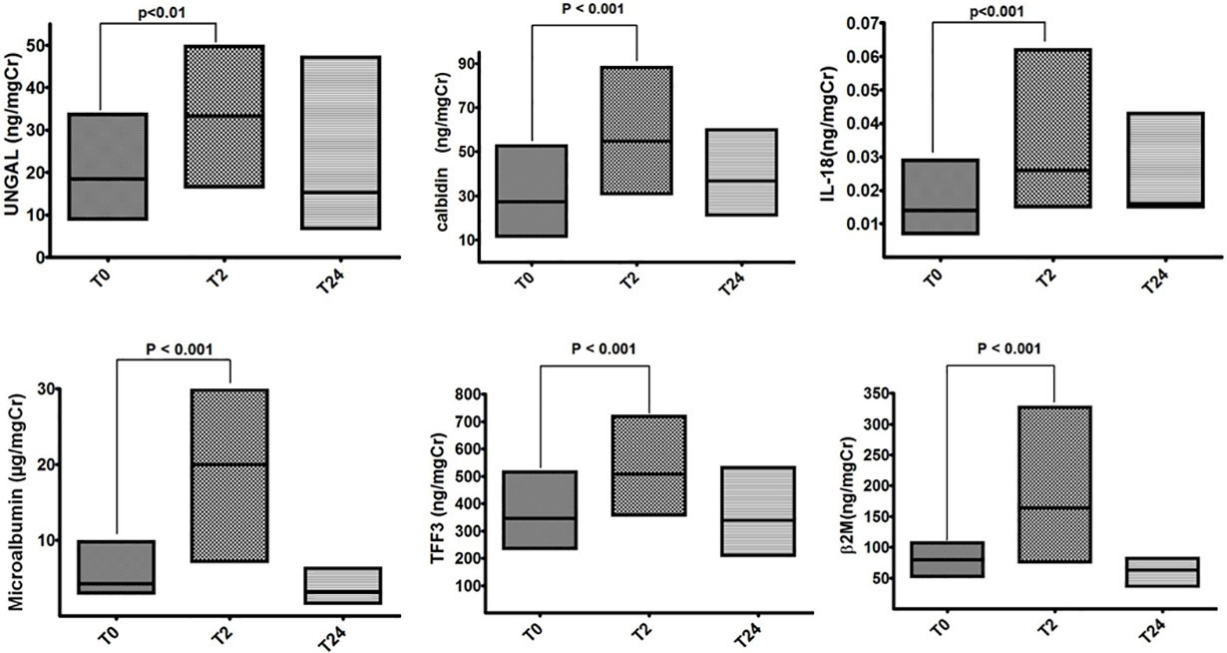
Box plots (median, 25% and 75% quartiles) of urinary biomarkers of kidney damage: neutrophil gelatinase-associated lipocalin (UNGAL), interleukin 18 (IL-18), calbindin, microalbuminuria (μalbum), trefoil factor-3 (TFF3) and β-2 microglobulin (β2M).

All assessed urinary renal function biomarkers increased significantly two hours after the exercise and returned to values similar to baseline 24 hours after the exercise (Fig 3 and Table 2). UNGAL increased in 70%, IL-18 in 74%, microalbuminuria in 87%, calbindin in 70%, TFF3 in 81% and β2M in 77% of the study participants.

When we separately analyzed the changes in the urinary biomarkers of men and women, the increases in IL-18, microalbuminuria, calbindin, TFF3 and β2M had a similar temporal pattern and were statistically significant for both genders.

UNGAL (ng/ml) increased significantly in women: 20.2 at baseline, 38.9 at 2 hours and 23.9 at 24 hours (p<0.05 baseline vs. two hours). Five women had UNGAL values greater than 100 ng/mgCr two hours after exercise. In men, UNGAL increased from 17.3 at baseline to 26.8 at two hours and decreased to 9.6 at 24 hours, but the difference was not statistically significant (p = 0.1004).

### Correlation between RPE and muscle and renal injury biomarkers

There was a significant and positive correlation between RPE and UNGAL (r = 0.3886, 95% CI 0.1371–0.5930, p = 0.0026) and RPE and microalbuminuria (r = 0.4632, 95% CI 0.2253–0.6490, p = 0.0003). There was no positive correlation between RPE and the other urinary kidney injury biomarkers. There was no correlation between RPE and CK and RPE and myoglobin.

## Discussion

This exploratory prospective cohort study showed that a single HIIRT session lasting four minutes caused an early and significant increase in biomarkers of muscle and renal injury. There was a positive and significant correlation between exercise intensity and both UNGAL and microalbuminuria. HIIRT had similar effects in both men and women, except for SCr and UNGAL. There are no prospective studies on the effects of resistance exercises on renal function available for comparison, but case reports of resistance exercises associated-AKI, including severe dialysis-dependent AKI and renal histology showing acute tubular necrosis after high-intensity resistance exercises have been described [11,16]. The limitations of this study are that we analyzed a single HIIRT session and had a short follow-up time.

In the present study, we found a modest but statistically significant increase in SCr 24 hours after the exercise only in men, and at least two of them had SCr increases greater than 0.3 mg/dl after 24 hours, which would be considered AKI by the KDIGO criteria. A small number of prospective studies have assessed the association between long-distance aerobic exercises with SCr increases compatible with AKI, as a study in marathon runners where 82% of the assessed participants met the criteria for AKI by the AKIN criteria [17].

There was a clear and statistically significant increase in UNGAL in the women. In both men and women, there were statistically significant increases in urinary IL-18, calbindin, μalbuminuria, TFF3 and β2M two hours after HIIRT. While there are no similar studies for comparison, there is evidence of acute increases in serum and urinary biomarkers of kidney injury in healthy long-distance runners, including serum cystatin C, albuminuria, urinary β2M, NGAL, KIM-1, IL-18, IL-6 and TNF-α. In fact, a prospective observational study of marathon runners found elevations in urinary albumin, NGAL and IL-18 of similar magnitude and kinetics (increased values detected within minutes after completing the running with a return to baseline after 24 hours) to those in the present study [17]. The elevation in biomarkers indicative of structural kidney injury and repair observed in the present study was below the levels usually found in patients with clinical AKI and are suggestive of the presence of kidney stress or mild kidney injury [18].

UNGAL values increased almost two times in our study, and in five women, the UNGAL reached values above 100 ng/mgCr, which is compatible with clinical AKI in some scenarios. NGAL, a small protein (25 kDa) of the lipocalin family, has a bacteriostatic function. Neutrophils, the uterus, the prostate, the salivary glands, the lungs, the trachea, the stomach, the colon, and the kidneys produce and express NGAL at consistent low levels [19]. The thick ascending limb of the loop of Henle and the intercalated cells of the collecting duct are likely the major sites of renal NGAL production [20]. NGAL produced in tissues other than the kidneys is filtered by the glomerulus and reabsorbed by the proximal tubules. Urinary NGAL levels in healthy individuals are usually around 20 ng/ml, which is consistent with the baseline level measured in the participants in the present study [20]. NGAL genes are rapidly expressed after renal insult, and a robust body of evidence has shown that urinary NGAL increases within minutes to a few hours after ischemic or toxic kidney injury. Because these NGAL characteristics have been shown to be early markers of renal structural damage [19,20], our findings are indicative of HIIRT-associated kidney injury, possibly at the thick ascending Henle loop, proximal tubule or both since the average baseline levels of the studied population were normal, and there are no other factors such as infection or increased extra-renal production of NGAL to confound the results. There was a significant positive correlation between exercise intensity measured by RPE and UNGAL, suggesting that the higher the exercise intensity more intense was the structural renal injury. The finding in this study that UNGAL was significantly increased only in women is consistent with the fact that its production is more elevated in females than in males [21].

IL-18 is a 24 kDa pleiotropic cytokine of the IL-1 family of cytokines, which controls innate and adaptive immunity [19]. IL-18 is constitutionally expressed in numerous tissues and cells, including monocytes, Kupffer cells, keratinocytes, osteoblasts, adrenal cortex cells, intestinal epithelial cells, microglial cells, synovial fibroblasts, proximal tubular epithelial cells, and the intercalated cells of the collecting ducts [19]. Increased levels of IL-18 usually occur during endogenous inflammatory processes, such as sepsis [22]. This cytokine is considered to be both a mediator and a biomarker of AKI [19], and IL-18 urinary levels increased few hours after exposure to nephrotoxic agents or ischemic injury in patients developing AKI. In the present study, we found an early increase in urinary IL-18 separate from other factors that might increase it systemically, such as infection. It is unlikely that the HIIRT session increased its plasma levels since the few studies assessing the effects of exercise on systemic IL-18 levels showed no acute changes and actually found decreased levels in the medium and long term [23,24]. Thus, the current findings suggest that the observed increase in urinary IL-18 is in fact attributable to renal tubular injury.

Increased microalbuminuria has been associated with endothelial dysfunction, systemic inflammation, glomerular injury, proximal tubule injury, chronic kidney disease and AKI [25,26] and has been reported after exercise [27]. In this study, the urinary excretion of albumin increased significantly immediately after the HIIRT session. The strenuous work out performed by the study participants probably caused this increase in albuminuria, a phenomenon previously reported with exhausting exercises and likely associated with renal and systemic vasoconstriction, acute inflammatory burst and oxidative stress [27]. There was an extremely significant positive correlation between exercise intensity measured by RPE and microalbuminuria, suggesting that the higher the exercise intensity more intense was the HIIRT-induced inflammatory burst.

Calbindin is a ubiquitous and small Ca^2+^-binding protein from the troponin C superfamily that functions as a calcium buffer and sensor. It is present in diverse tissues and cells, including the neuronal sub-populations in the central and peripheral nervous systems, enteric neuroendocrine cells and renal distal tubular cells. Urinary calbindin concentrations increase rapidly after renal injury in septic and nephrotoxic animal models and return to baseline levels 24 hours after insult [28,29]. There are few studies assessing calbindin clinically, but its levels increased eight-fold three days after an infusion of cisplatin in patients with solid tumors. Its increase in the present study suggests renal distal tubule injury.

TFF3 is a 13.1 kDa peptide that is part of the trefoil factor family (comprising TFF1, TFF2 and TFF3) that is expressed in the goblet cells of the intestines, the colon and the kidneys. In humans, TFFs are produced throughout the urinary tract, and TFF3 predominates in cells of the proximal and distal tubules and collecting ducts [30]. Members of the TFF family play a pivotal role in the protection and repair of epithelial cells and mucosa, mainly within the gastrointestinal system. More recently, TFF3 has been considered to be a part of the regenerative defenses of the kidney, and a biomarker of acute and chronic kidney injury [31–33]. In animal models, changes in urinary levels of TFF3 are associated with AKI [34]. An ad hoc study of the Atherosclerosis Risk in Communities (ARIC) Study and the ARIC Carotid MRI suggested that elevated TFF3 urinary levels might be indicative of ongoing repair of the renal tubular epithelium and inflammation [32]. Initial studies in renal transplant patients showed that TFF3 levels rise instantly after transplantation and then drop for up to seven days, regardless of the occurrence of delayed graft function [34]. The rapid increase in TFF3 in the present study is likely due to HIIRT-induced proximal and distal tubular injury, and it might be associated with the beginning of the epithelial regenerative process. A prospective observational study including 22 marathon runners also found an early increase in repair biomarkers after exercise (human cartilage glycoprotein 39—YKL-40 and monocyte chemoattractant protein 1—MCP-1) [17].

β2M, a 12 kDa protein continuously generated by all nucleated cells of the body, is freely filtered by the glomeruli and almost totally (99.9% of the filtered protein) reabsorbed and catabolized in the proximal convoluted tubule. The urinary concentration of β2M increases when glomerular and/or tubular injury occurs [35] and its ability to detect nephrotoxin-induced tubular injury have been shown in different clinical scenarios [36]. In the context of the present study, the increased urinary β2M likely reflects proximal tubule injury.

Strenuous exercise-associated acute renal damage and AKI might occur due to different mechanisms evoked by the exercise, including rhabdomyolysis, systemic and renal vasoconstriction, cardiac dysfunction, and a systemic inflammatory burst and oxidative stress. These triggers might be associated with individual aggravating risk factors, such as dehydration, age, habitual level of physical activity, presence of genetic myopathies, use of nonsteroidal anti-inflammatory drugs, alcohol abuse, use of recreational drugs, high environmental temperature, presence of infectious illness, and intensity of the exercise.

In the current study, HIIRT induced early and progressive muscle injury, as shown by the muscle soreness, and significant changes in serum CK and myoglobin in the majority of the individuals assessed, with CK elevation indicative of rhabdomyolysis in 19% of the participants. Rhabdomyolysis is a well-known potential cause of AKI due to myoglobin nephrotoxicity, heme-induced renal vasoconstriction, systemic inflammation, and oxidant injury of the renal tissue through heme-induced reactive oxygen species [37]. There are previous studies relating the development of strenuous exercise-associated muscle breakdown or rhabdomyolysis, in association with clinical AKI or SCr increase compatible with AKI diagnosis in prospective studies of long-distance running, triathlons and duathlons, and case reports of spinning, HIIT, HIIRT, weight lifting and resistance exercises. However, there is no previous prospective study assessing this association after HIIRT, as described in the present manuscript. In addition, European epidemiological studies found that the number of patients admitted to the hospital with exercise-induced rhabdomyolysis increased strikingly from 2010–2011 to 2014–2015. The majority of patients were referred as a result of strength training at fitness centers, with up to 6% of the patients developing AKI. The authors hypothesized that this observed growth in the frequency of exercise-induced rhabdomyolysis might be due to the culture of high-intensity training and increased societal pressure to exercise [38,39]. In some instances, severe strenuous exercise-induced rhabdomyolysis characterized by extremely high CK levels was reported without SCr increases [12,13]. However, in practically all cases with increased CK and normal SCr, the authors did not assess biomarkers indicative of structural kidney injury. It is conceivable that severe rhabdomyolysis might promote subclinical kidney injury and mild kidney structural injury without causing a detectable change in the glomerular filtration rate. Consistent with this hypothesis, a recent publication described a group of eight military recruits hospitalized with severe exertional rhabdomyolysis with normal values of SCr and cystatin C but significant increases in serum NGAL, suggesting renal stress, as occurred in the present study [40].

What is the clinical significance of the present findings? The individuals participating in our study were healthy, young, and regular exercisers, and they did not have overt risk factors for kidney injury. However, HIIRT caused muscle damage (breakdown and rhabdomyolysis) and subclinical kidney injury in the participants. This situation potentially put these individuals in a more vulnerable position for clinically significant kidney injury, if risk factors for AKI such as dehydration, systemic infection, use of NSAIDs or others occur concurrently. Similarly, the long-term effects of repetitive weekly bursts of subclinical injury in individuals practicing high-intensity exercises are unknown. However, it is also possible that some individuals may develop improved resilience to renal insults, as observed with remote ischemic preconditioning preventing AKI, which enhanced the renal recovery in patients with AKI and reduced the long-term incidence of major adverse kidney events in high-risk patients undergoing cardiac surgery [41]. Likewise, acute exercise or continuous training schedules may induce or activate heat shock proteins, a group of proteins that is crucial in the preservation of protein and cell homeostasis [42]. A limited amount of evidence has shown an early increase in urinary biomarkers of kidney repair and enhancement of blood levels of heat shock protein (HSP70) after strenuous exercise [42].

In conclusion, a single session of HIIRT in young and healthy individuals caused muscle damage and renal injury. Some individuals had biomarker levels that would be compatible with an AKI diagnosis in clinically relevant scenarios. High-intensity exercises must be practiced under professional supervision in a gradual and adequate manner for individuals initiating regular physical activity, who should be warned about the possibility of rhabdomyolysis and instructed to avoid modifiable AKI risk factors. Our results suggest that high-intensity exercises must be practiced with extreme caution or avoided in individuals with non-modifiable high risks for kidney injury.

## Supporting information

S1 File (Table A) Characteristics of the study participants. (Table B) Assessment of muscle and kidney injury biomarkers at baseline and at 2 and 24 hours after exercise.
(DOCX)
