# Supplementary material for: High intensity resistance training causes muscle damage and increases biomarkers of acute kidney injury in healthy individuals
Source: PLoS One. 2018 Nov 6;13(11):e0205791. doi: 10.1371/journal.pone.0205791 (PMC6219767; doi:10.1371/journal.pone.0205791)
Supplement: S1 File — (Table A) Characteristics of the study participants. (Table B) Assessment of muscle and kidney injury biomarkers at baseline and at 2 and 24 hours after exercise. (DOCX) [file pone.0205791.s001.docx]

**S1 file**

**Table A.**  Characteristics of the study participants

|  | Men (n=29) | Women (n=29) | All (n=58) |
| --- | --- | --- | --- |
| Age | 24 (22-28) | 24 (21-28) | 24 (21-28) |
| Weight (kg) | 77.3±12.0 | 61.3±9.7 | 69.3±13.5 |
| Height (cm) | 176 (172-182) | 163 (158 a 165) | 168 (162-176) |
| BMI (kg/m^2)^ | 24.5±2.8 | 23.3±3.4 | 23.9±3.1 |

Data are the median (25% and 75% quartiles) or mean±SD; BMI: body mass index.

**Table B.** Assessment of muscle and kidney injury biomarkers at baseline and at 2 and 24 hours after exercise

|  | **Baseline (T0)** | **2 hours after HIIRT (T2)** | **24 hours after HIIRT (T24)** | **p** |
| --- | --- | --- | --- | --- |
| **Muscle injury markers** |  |  |  |  |
| CK (IU/L) | 123 [102-172] | 158 [124-238]*^a^* | 340 [196-4906]*^b,c^* | <0.0001 |
| Myoglobin (ng/ml) | 21.0 [21.0 – 29.8] | 88.6 [62.9 – 206.6]*^a^* | 39.1 [23.6 – 93.6]*^b^* | <0.0001 |
| **Serum kidney injury marker** |  |  |  |  |
| SCr (mg/dl) | 0.91±0.17 | 0.90±0.17 | 0.94±0.23^d^ | 0.0274 |
| **Urine kidney injury markers** |  |  |  |  |
| UNGAL (ng/mgCr) | 18.5 [8.9–33.7]) | 33.4 [16.6–49.7]*^e^* | 15.3 [6.8–47.2] | <0.0001 |
| IL-18 (ng/mgCr) | 0.014 [0.007–0.029] | 0.026 [0.015–0.062*^a^* | 0.015 [0.016–0.043] | 0.0003 |
| µalbumin (µg/mgCr) | 4.3 [3.0–9.8] | 20.0 [7.2–29.9] *^a^* | 3.2 [1.6–6.3] | <0.0001 |
| Calbindin (ng/mgCr) | 27.4 [11.1–52.6] | 54.8 [30.9–88.1] *^a^* | 36.8 [21.2–59.9] | 0.0003 |
| TFF3 (ng/mgCr) | 347 [234-5162] | 508 [357-719] *^a^* | 339([210-531] | <0.0001 |
| β2M (ng/mgCr) | 80 [52-107] | 164 [76-327] *^a^* | 63 [36-82] | <0.0001 |

Values are the mean±SD or median (25-75% quartiles); HIIRT: high-intensity resistance training; RPE: rating of perceived exertion; CK: creatine phosphokinase; SCr: serum creatinine; UNGAL: urinary neutrophil gelatinase-associated lipocalin; IL-18: interleukin 18; TFF3: trefoil factor-3; β2M: β-2 microglobulin. ^a^ vs. T0 p<0.001; ^b^ vs. T0 p<0.001; ^c^ vs. T2 p<0.001; ^d^ vs. T2 p<0.05; ^e^ vs. T0 p<0.01.

.
